# Effect of statins on experimental postoperative adhesion: a systematic review and meta-analysis

**DOI:** 10.1038/s41598-018-33145-z

**Published:** 2018-10-03

**Authors:** Geun Joo Choi, Hee Kyung Park, Dong Su Kim, Donghyun Lee, Hyun Kang

**Affiliations:** 1 Department of Anesthesiology and Pain Medicine, College of Medicine, Chung-Ang University, 84 Heukseok-ro, Dongjak-gu, Seoul, 06911 Republic of Korea; 2 Department of Biomedical Engineering, School of Integrative Engineering, Chung-Ang University, 84 Heukseok-ro, Dongjak-gu, Seoul, 06911 Republic of Korea

## Abstract

Adhesion is a significant concern after surgery. Many researchers studied the anti-adhesive effect of statin, of which results were inconsistent. Thus, we purposed to perform a systematic review and meta-analysis to evaluate the effect of statins on postoperative adhesion in an experimental study. A comprehensive search was conducted using MEDLINE, EMBASE, and Google Scholar to identify animal studies that investigated the postoperative anti-adhesive effect of statins applied at the surgical area. Primary outcome measure was gross adhesion score. Secondary outcomes included microscopic adhesion score and tissue plasminogen activator (t-PA) activity. Totally, 298 rats from 9 animal studies (172 rats received statin therapy and 126 rats received placebo or no treatment) were included in the final analysis. The combined results showed that gross and microscopic adhesion scores were significantly lower in the statin group in comparison to the control group (standardized mean difference [SMD] = 1.65, 95% confidence interval [CI]: 1.02 to 2.28, P_chi_^2^ < 0.001, I^2^ = 77.9%; SMD = 1.90, 95% CI: 1.10 to 2.79, P_chi_^2^ < 0.001, I^2^ = 84.5%, respectively). However, there was no evidence of a difference in t-PA activity (SMD = −3.43, 95% CI: −7.95 to 1.09, P_chi_^2^ < 0.001, I^2^ = 95.5%). In conclusion, statins were effective in preventing postoperative adhesion, as assessed based on gross and microscopic adhesion scores in rats.

## Introduction

Postoperative adhesion formation remains a major cause of morbidities such as bowel obstruction, infertility, and subsequent persistent pain^1–4^. Furthermore, subsequent adhesion-related hospital readmissions and reoperations impose a significant social and economic burden^5^.

Various strategies have been employed for the prevention of postoperative adhesion formation including abdominal tissue manipulation and irritation reduction^5^, mechanical barriers such as different film types^6^, solutions or different gel types^7^, chemical barriers such as heparin^8^, non-steroidal anti-inflammatory agents^9^, fibrinolytic agents^10^, thrombin-activated fibrinolysis inhibitors^11^, and a combination of mechanical and chemical barriers^12^. However, no method has proven to prevent adhesion consistently and completely.

Despite the use of anti-adhesive agents, postoperative adhesion rates remain high^13^.

Adhesion formation is triggered by trauma to the surgery area. Surgical trauma induces an inflammatory response that leads to activation of the extrinsic pathway of the coagulation cascade, resulting in fibrosis^14^. Under normal conditions, fibrosis is resolved by fibrinolysis. The process of fibrinolysis is mediated by the enzyme plasmin, which is derived from its inactive substrate plasminogen via tissue-type plasminogen activator (t-PA). t-PA is inhibited by plasminogen activator inhibitor-1 (PAI-1)^15,16^. However, under ischemic or inflammatory conditions such as surgery, the fibrinolytic system is suppressed and fibroblasts assemble into dense adhesions^17^. This, in turn, disturbs the balance between t-PA and PAI-1, resulting in increased adhesion formation^18^.

The family of statins, 3-hydroxy-3-methylglutaryl coenzyme A (HMG-CoA) reductase inhibitors, has largely been used in patients with atherosclerotic disease and hyperlipidemia. Various experimental studies have shown that statins also have antioxidant, anti-inflammatory, and pro-fibrinolytic properties^19^, all of which may play a role in the process of adhesion formation and prevention. Thus, statins theoretically have the potential to prevent postoperative adhesion.

Though several studies have investigated the anti-adhesive effect of statins following surgery, the results have been inconsistent. There are currently no systematic review and meta-analysis on the postoperative anti-adhesive effects of statins. Therefore, our objective was to identify and summarize the currently available data from animal studies investigating the anti-adhesive effects of statins after surgery.

## Results

Sixty-eight records were found using OVID-MEDLINE, EMBASE, and Google Scholar and 6 were identified through manual searches. After adjusting for duplicates, 71 studies remained. Of these, 58 studies that did not align with the aim of our meta-analysis were excluded following a review of the titles and abstracts. The full texts of the remaining 13 studies were reviewed in detail, and 4 studies were excluded for the following reasons: oral use of statins (n = 3)^20–22^ and use in clinical setting (n = 1)^23^.

**Figure 1:**
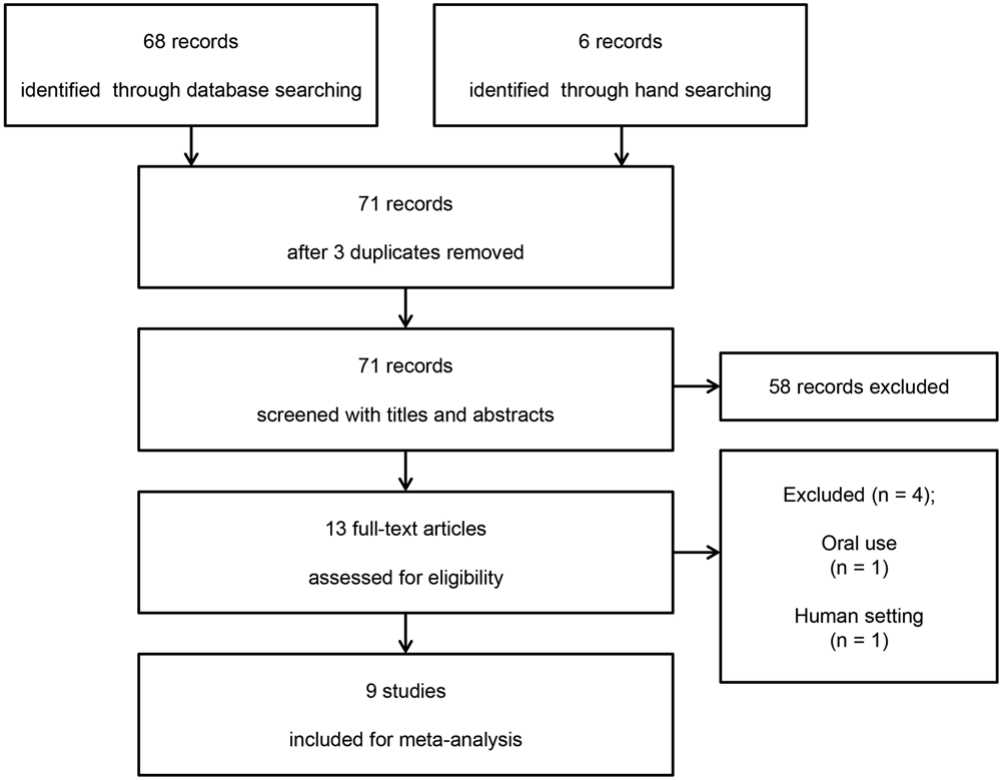
Flow diagram showing the number of abstracts and articles identified and evaluated during the review process.

Thus, 9 studies with a total of 298 rats (172 rats received statin therapy and 126 rats received placebo or no treatment) met the inclusion criteria and were included in this systematic review and meta-analysis (Fig. 1).

### Study characteristics

The characteristics of the included studies are shown in Table 1. The studies investigated the effects of simvastatin^24–26^, atorvastatin^27–29^, rosuvastatin^30,31^, and lovastatin and atorvastatin^17^. The surgeries performed included laparotomy (cecum)^17,24,26,28,29^, laparotomy (uterine)^27^, femoral condyle surgery^30^, and laminectomy^25,31^. Male Wistar rats^17,24,26,28,29,31^, female Wistar rats^27^, and male Sprague-Dawley rats^25,30^ were used.

**Table 1:** Characteristics of Included Studies.

First author, publication year,	Animal	Surgery	Group	Definition
Javaherzadeh, 2016	Male Wistar albino rats	Laparotomy (cecum)	Control	N/S
			Experimental	N/S + Simvastatin (30 mg/kg)
Yilmaz, 2009	Non-pregnant, female Wistar albino rats	Laparotomy (uterine horns)	Control	No treatment
			Low dose atorvastatin	2.5 mg/kg/day atorvastatin
			High dose atorvastatin	30 mg/kg/day atorvastatin
			Metformin	50 mg/kg/day metformin
Lalountas, 2012	Male Wistar rats	Laparotomy (caecum)	No film	No treatment
			Placebo	Carboxymethylcellulose film without atorvastatin
			Low-dose group	Statofilm containing 0·125 mg/kg atorvastatin
			High-dose group	Statofilm containing 1 mg/kg atorvastatin
Lalountas, 2010	Male Wistar rats	Laparotomy (cecum)	Group 1	No treatment
			Group 2	Atorvastatin 30 mg/kg
			Group 3	HA/CMC membrane
			Group 4	HA/CMC membrane + Atorvastatin 30 mg/kg
Sun, 2015	Male Sprague-Dawley rats	Laminectomy	Control	No treatment
			Chitosan	Chitosan
			Simvastatin	Simvastatin (1 mg/ml)
Aaron, 2007	Male Wistar rats	Laparotomy (caecum)	Control	Control
			Lovastatin	Lovastatin (30 mg/kg)
			Atrovastatin	Atorvastatin (30 mg/kg)
Kucuk, 2007	Male Wistar rats	Laparotomy (caecum)	Group 1	Simvastatin 0.57 mg/kg/day injected intraperitoneally right after operation and 5 day after
			Group 2	Simvastatin 0.57 mg/kg/day injected via gavage right after operation and 5 day after
			Group 3	N/S
Wu, 2016	Male Sprague-Dawley rats	Femoral condyle exposing surgery	saline control group	Gelatin sponges soaked with N/S
			ROS 10 mg/kg group	Gelatin sponges soaked with 10 mg/kg of rosuvastatin
			ROS 20 mg/kg group	Gelatin sponges soaked with 20 mg/kg of rosuvastatin
Gűrer, 2015	Male Wistar rats	Laminectomy	Group 1	Laminectomy
			Group 2	Spongostan
			Group 3	Spongostan soaked with 20 mg/Kg rosuvastatin
			Group 4	Systemic 20 mg/Kg rosuvastatin

N/S: normal saline.

Three studies with multiple groups had two eligible groups for comparison^25,26,31^ and two studies with multiple groups had three eligible groups for comparison^27,28^, in which only eligible groups were selected and included in our meta-analysis. In one study with four groups^29^, we produced two sub-studies having two each groups depending on whether a sodium hyaluronate/carboxymethylcellulose membrane was applied to both statin and control groups, or not. In two sub-studies, two independent investigations were performed. In four studies^17,27,28,30^, there were two eligible statin groups comparing control group, respectively. We combined the two statin groups to create a single pair-wise comparison to avoid the unit-of-analysis error.

### Gross adhesion score

**Figure 2:**
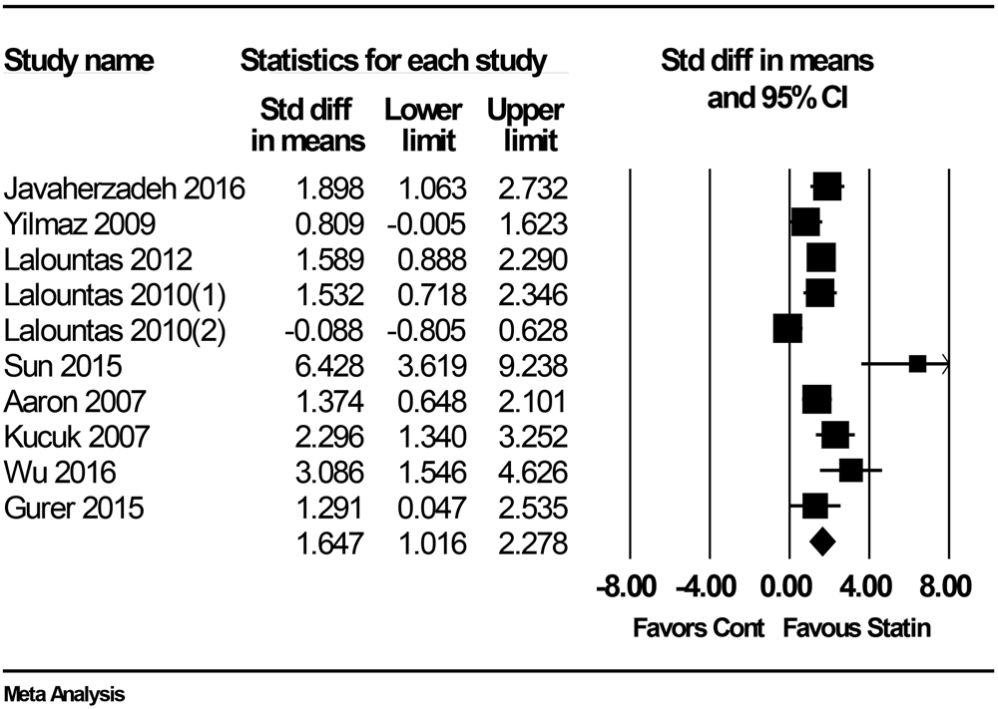
Forest plot showing gross adhesion score. M-H: Mantel-Haenszel.

Gross adhesion scores were reported in all studies. These scores were reported on a 5-point scale^24,26,28^, 4-point scale^25,30^, or 4-point and 5-point scales^27,29^ and number of ischemic buttons with attached adhesions^17^. The definitions for gross adhesion score in each study are described in Table 2. The combined results showed that gross adhesion score was significantly lower in the statin group (SMD = 1.65, 95% CI: 1.02 to 2.28, P_chi_^2^ < 0.001, I^2^ = 77.9%; Fig. 2).

**Table 2:** Definition of gross and microscopic adhesion scores.

First author, publication year	Gross adhesion score	Microscopic adhesion score
Javaherzadeh, 2016	0: No adhesion, 1: One adhesion band, no vessel, easily separated, 2: Two thin adhesion bands, no vessel, easily separated, 3: Three thin adhesion bands, no vessel, easily separated, 4: More than three thin adhesion bands, easily separated with no vessel or diffuse adhesion bands with vessels	0: No adhesion, 1: Fat, 2: Fat and fibrosis, 3: Fibrosis
Yilmaz, 2009	0: no uterine adhesion, 1: 1–25% involvement, 2: 26–50%, 3: 51–75%, 4: 76–100%	0: no fibrosis, 1: minimal, loose, 2: moderate, 3: florid dense
	0: no adhesion, 1: filmy avascular, 2: vascular or opaque, 3: cohesive attachment of uterine horn to each other or other abdominal organs	
	0: no adhesion, 1: the adhesion could be separated from tissue with gentle traction, 2: the adhesion could be separated from tissue with moderate traction, 3: requiring sharp dissection	
Lalountas, 2012	0: No adhesions, Single band, between viscera, or from one viscus to abdominal wall, Single band, between viscera, or from one viscus to abdominal wall, 1: Single band, between viscera, or from one viscus to abdominal wall, 2: Two bands between viscera or from viscera to abdominal wall, 3: More than two bands between viscera, or from viscera to abdominal wall, or intestinal loop forming a mass without being adherent to abdominal wall, 4: Viscera directly adherent to abdominal wall, irrespective of number and extent of adhesive bands	0: none, 1: slight, 2: moderate, 3: severe
	0: Complete absence of adhesions, 1: Single band of adhesions, between viscera, or from 1 viscus to abdominal wall, 2: Two bands: between viscera or from viscera to abdominal wall, 3: More than 2 bands: between viscera, or viscera to abdominal wall, or whole of intestines forming a mass without being adherent to abdominal wall, 4: Viscera directly adherent to abdominal wall, irrespective of number and extent of adhesive bands	
Lalountas, 2010	0: No adhesions, Single band, between viscera, or from one viscus to abdominal wall, Single band, between viscera, or from one viscus to abdominal wall, 1: Single band, between viscera, or from one viscus to abdominal wall, 2: Two bands between viscera or from viscera to abdominal wall, 3: More than two bands between viscera, or from viscera to abdominal wall, or intestinal loop forming a mass without being adherent to abdominal wall, 4: Viscera directly adherent to abdominal wall, irrespective of number and extent of adhesive bands	
	0: No adhesion, 1: Filmy thickness, avascular, 2: Limited vascularity, moderate thickness, 3: Well vascularized, dense thickness	
SUN, 2015	Grade 0: epidural scar tissue was not adherent to the dura mater, Grade 1: epidural scar tissue was adherent to the dura mater, but easily dissected, Grade 2: epidural scar tissue was adherent to the dura mater and difficultly dissected without disrupting the dura matter, Grade 3: epidural scar tissue was firmly adherent to the dura mater, and could not be dissected	Number of fibroblast using a light microscope at a magnification of 400
Aaron 2007	percent adhesion score based on the number of ischemic buttons with attached adhesions	
Kucuk, 2007	0: No adhesions, Single band, between viscera, or from one viscus to abdominal wall, Single band, between viscera, or from one viscus to abdominal wall, 1: Single band, between viscera, or from one viscus to abdominal wall, 2: Two bands between viscera or from viscera to abdominal wall, 3: More than two bands between viscera, or from viscera to abdominal wall, or intestinal loop forming a mass without being adherent to abdominal wall, 4: Viscera directly adherent to abdominal wall, irrespective of number and extent of adhesive bands	
Wu, 2016	0, no adhesion; 1, weak, mild, filmy adhesions that can be easily eliminated by manual traction; 2, moderate adhesions that were able to be eliminated by manual traction; 3, dense and firm adhesions that had to be surgically removed	Number of fibroblast using a light microscope at a magnification of 200
Gűrer, 2015	Grade 0: epidural scar tissue was not adherent to the dura mater, Grade 1: epidural scar tissue was adherent to the dura mater, but easily dissected, Grade 2: epidural scar tissue was adherent to the dura mater and difficultly dissected without disrupting the dura matter, Grade 3: epidural scar tissue was firmly adherent to the dura mater, and could not be dissected	Grade 0: dura mater is free of scar tissue, Grade 1: only thin fibrous bands are observed between the scar tissue and the dura mater, Grade 2: continuous adherence is observed in less than two-thirds of the laminectomy defect, Grade 3: scar tissue adherence is large, affecting more than two-thirds of the laminectomy defect, or the adherence extended to the nerve roots.

**Figure 3:**
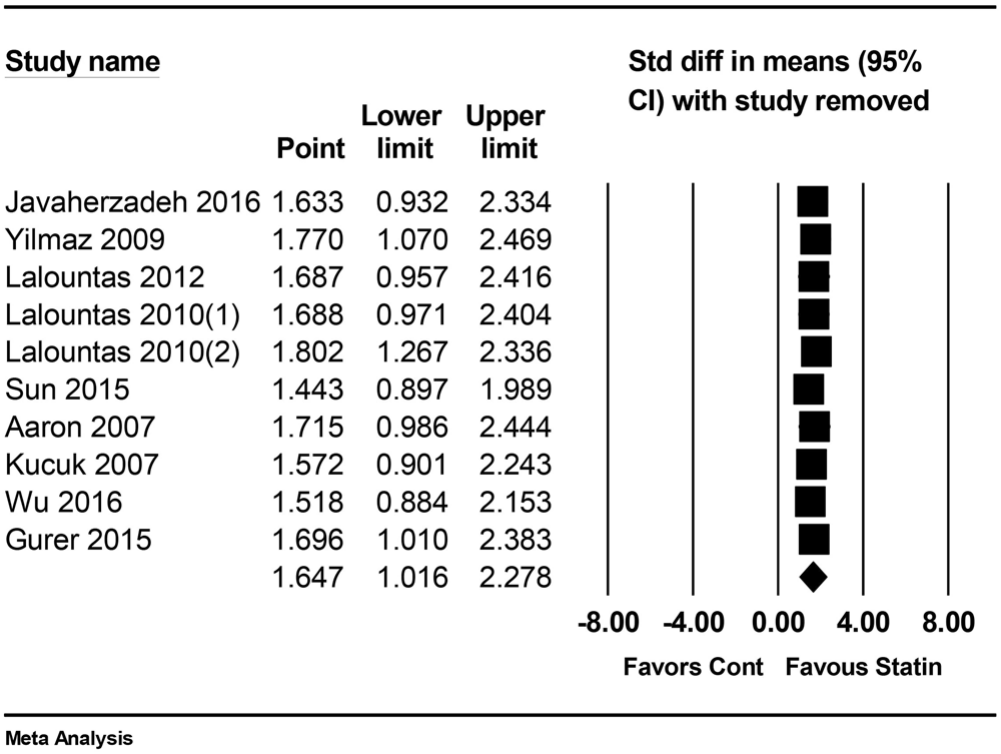
Sensitivity analysis excluding one study at a time for gross adhesion score. M-H: Mantel-Haenszel.

As the result of subgroup analysis according to the surgery type, gross adhesion score was significantly lower in statin group for laparotomy (SMD 1.32, 95% CI: 0.74 to 1.90, P_chi_^2^ = 0.001, I^2^ = 73.8%). For laminectomy, there was no significant difference (SMD 3.70, 95% CI: −1.33 to 8.72, P_chi_^2^ < 0.001, I^2^ = 90%), but the values of effect size and I^2^ increased. Sensitivity analyses through the sequential removal of one study at a time did not alter the significance (Fig. 3).

### Fibrosis microscopic adhesion score

**Figure 4:**
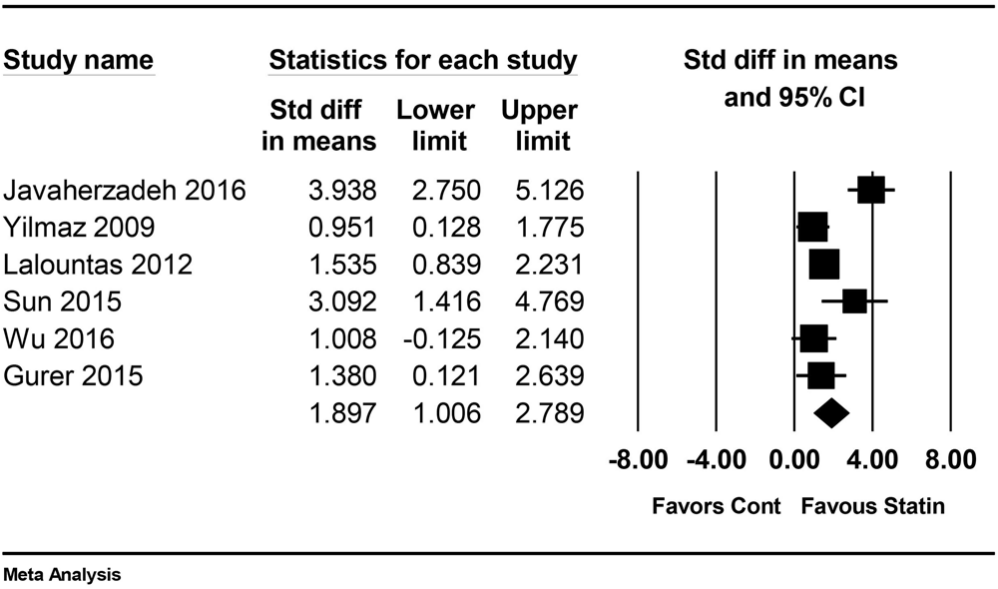
Forest plot showing microscopic adhesion score. M-H: Mantel-Haenszel.

Fibrosis microscopic adhesion scores were reported in 6 studies^24,25,27,28,30,31^. The definitions for microscopic adhesion score used in each study are described in Table 2. The combined results showed that microscopic adhesion score was significantly lower in the statin group than in the placebo group (SMD = 1.90, 95% CI: 1.01 to 2.79, P_chi_^2^ = 0.001, I^2^ = 76.6%; Fig. 4).

As a result of subgroup analysis according to the surgery type, microscopic adhesion score was significantly lower in statin group for both laparotomy and laminectomy, respectively (SMD 2.07, 95% CI: 0.57 to 2.80, P_chi_^2^ = 0.122, I^2^ = 52.4% in laparotomy; SMD 2.14, 95% CI: 0.48 to 3.81, P_chi_^2^ = 0.110, I^2^ = 61% in laminectomy). Sensitivity analyses by removing one study at a time did not change the significance of the results.

### t-PA activity

**Figure 5:**
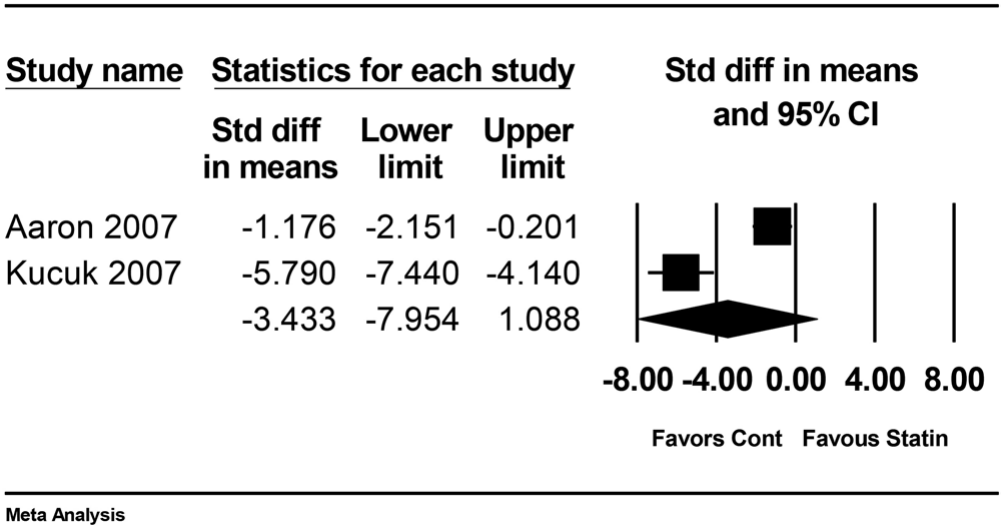
Forest plot showing t-PA activity. M-H: Mantel-Haenszel.

t-PA activity was reported in two studies^17,26^. The combined results showed no evidence of a difference (SMD = −3.43, 95% CI: −7.95 to 1.09, P_chi_^2^ < 0.001, I^2^ = 95.5%; Fig. 5).

### Methodological quality and publication bias

A summary of the methodological quality assessment for each study is shown in Table 3. The methodological quality scores ranged from 3 to 5, with 5 studies scoring 4 or 5 points. Publication bias was not analyzed since the number of included studies was less than 10.

**Table 3:** Assessment of methodological quality.

First author, publication year,	Statement of random allocation	Husbandry conditions	Compliance with animal welfare regulations	Potential conflict of interest	Peer reviewed	Score
Javaherzadeh, 2016	0	0	1	1	1	3
Yilmaz, 2009	0	1	1	1	1	3
Lalountas, 2012	1	1	1	1	1	5
Lalountas, 2010	1	1	1	1	1	5
Sun, 2015	0	0	1	1	1	3
Aaron, 2007	0	1	1	1	1	4
Kucuk, 2007	0	0	1	1	1	3
Wu, 2016	1	1	1	1	1	5
Gűrer, 2015	1	0	1	1	1	4

Methodological quality was assessed based on statements of 1) random allocation into treatment and control groups, 2) husbandry conditions (e.g., light/dark cycle, temperature, access to water, and environmental enrichment), 3) compliance with animal welfare regulations, and 4) potential conflicts of interests, and whether the study appeared in a peer-reviewed publication. Each article was assessed independently by two reviewers and scored on a scale from 0 to 5 points.

## Discussion

In the present study, statins applied at the surgical area reduced postoperative adhesion with respect to the evaluation of macroscopic adhesion and microscopic fibrosis scores. t-PA activity during the postoperative period was lower in the statin group, although this difference was not statistically significant.

Postoperative adhesions remain a significant problem for patients and surgeons as they can cause bowel obstruction, infertility, subsequent persistent pain, reoperation, and hospital readmissions^1–4^. The presence of adhesions has also been reported to be the greatest risk factor for bowel resection in patients with small bowel obstruction^32^. Furthermore, postoperative adhesion imposes a significant social and economic burden^5^; costs reportedly exceed $1 billion in the United States alone annually^33^.

To prevent or reduce postoperative adhesion, various strategies have been investigated: (1) techniques to reduce tissue manipulation and irritation^5^, (2) mechanical barriers^6,7^, (3) chemical barriers^8–11^, and (4) a combination of mechanical and chemical barriers^12^. However, none of these strategies have proven to be consistently and completely effective. One study reported that despite the use of anti-adhesive agent and improvement in surgical techniques, the overall incidence of adhesion-related readmissions has not diminished^13^. In this study, despite the use of Seprafilm, postoperative adhesion was prevented in only 51% of patients, and dense adhesions formed in 15% of patients^13^. Further, the cumulative risk of adhesive small bowel obstruction following abdominal surgery and readmission risk due to adhesion following colorectal surgery remain high (approximately 30%)^34,35^.

Adhesion formation is induced by trauma to the surgical area, initiating an inflammatory response. This inflammatory response activates the extrinsic pathway of the coagulation cascade via up-regulation of the expression of tissue factors by macrophages and mesothelial cells, resulting in the formation of a fibrin-rich inflammatory exudate^14^. Although fibrinolysis can resolve these fibrin bands under normal conditions, fibrinolysis is reduced under the ischemic or inflammatory conditions that accompany local damage to the surgically injured area. The suppression of fibrinolysis facilitates the infiltration of inflammatory cells and fibroblasts into the fibrin bands, causing the bands to organize into persistent dense adhesions^17^. The process of fibrinolysis, primarily the degradation of fibrin bands, is driven by the enzyme plasmin, which is converted from its inactive substrate plasminogen by t-PA and urokinase-type plasminogen activator (u-PA). Of those, tPA is the primary plasminogen activator synthesized in the abdomen by mesothelial cells and is responsible for 95% of the plasminogen conversion. tPA is inactivated by PAI-1 and PAI-2^15,16^. Intra-abdominal surgery can cause local damage to the peritoneum and induce inflammatory and ischemic conditions. It has also been observed to decrease t-PA and increase PAI-1 and PAI-2, resulting in inadequate peritoneal fibrinolysis and increase in fibrin exudates and adhesion formation^18^.

The family of statins, 3-hydroxy-3-methylglutaryl coenzyme A (HMG-CoA) reductase inhibitors, which catalyze the rate-limiting step in hepatic cholesterol, has been largely used in patients with atherosclerotic disease and hyperlipidemia. They also reportedly possess antioxidant, anti-inflammatory, and pro-fibrinolytic properties^19^, all of which may play a role in the process of adhesion formation and prevention. Further, statins have been shown to be potent modulators of fibrinolysis under both normal and inflammatory conditions^36^.

The fibrinolytic effects of statins have been demonstrated in human peritoneal mesotheslial cells^36,37^, human vascular smooth muscle cells^38^, and rabbit renal mesangial cells^39^. Statins were reported to stimulate fibrinolytic activity by significantly increasing t-PA levels and reducing PAI-1 levels^37^. Thus, statins theoretically have the potential to prevent postoperative adhesion. The findings in our meta-analysis confirmed the favorable effect of statins with regard to gross adhesion and microscopic fibrosis scores. However, inconsistent results exist in the literature regarding the oral administration of statins for the prevention of adhesion formation. Some experimental studies reported that oral simvastatin or fluvastatin reduced fibrosis in rotator cuff tears or laparotomy, respectively^21,22^, whereas there was studies reporting that oral simvastatin did show no effect on the fibrinolytic pathway in rats and human^20,40^.

The precise mechanism of postoperative adhesion is not clear yet. One of the suggested mechanism by which statins function to prevent intra-abdominal adhesion is increased t-PA and reduced PAI-1 levels. Aarons *et al*. reported that the intraperitoneal administration of atorvastatin and lovastatin significantly increased t-PA and mRNA levels and t-PA activity in the peritoneum^17^. Additionally, Kucke *et al*. reported that reduced adhesion was associated with increase in t-PA levels in the abdominal cavity^26^. Haslinger *et al*. demonstrated that simvastatin enhanced the fibrinolytic capacity of the human mesothelial cells at peritoneum where t-PA was activated and PAI-1 production was inhibited^37^. They reported that geranylgeranyl-modified intermediates and actin skeleton perturbation were mediated in this mechanism^37^. However, our meta-analysis could not confirm the association between t-PA activity and statins. This might be due to the lack of studies, since only two studies reported on t-PA activity. However, it is also possible that t-PA was not involved or bypassed the mechanism by which statins operate to prevent adhesion. Although the role of t-PA has been strongly implicated, in clinical practice, fibrinolytic agents and thrombin-activated fibrinolysis inhibitors continue to be suggested as chemical barriers, resulting in inconsistent outcomes^11,41^.

In terms of pathophysiology of epidural adhesion after laminectomy, there is a bit different point from abdominal adhesion. Songer *et al*. suggested that the replacement of epidural fat by a hematoma would cause epidural fibrosis^4^. Some researchers reported that a posterior invasion of fibroblast from the erector spinal muscle would cause peridural and epidural adhesion after laminectomy^42^. Most surgery type was laparotomy in included studies in our meta-analysis, but laminectomy also was performed in two studies. Hence, we conducted subgroup analysis according to surgery type, laparotomy and laminectomy. The significance of the results from macroscopic and microscopic adhesion scores did not change for laparotomy. On the other hand, macroscopic score was comparable for laminectomy although microscopic score was still higher in statin group. Epidural fibrosis after laminectomy should be based on both gross and microscopic evaluation, while macroscopic evaluation for intra-abdominal fibrosis after laparotomy may have a larger portion compared to microscopic evaluation. Given this, we suggest that statin can be beneficial for the adhesion following laminectomy as well as laparotomy.

We expect that our findings from this meta-analysis of animal studies will present the possibility and necessity of clinical research regarding anti-adhesive effect of statin, especially application of statin to the surgical site. Indeed, there are many trials to develop materials containing statin applying surgical site^43^, which would be actually beneficial for the prevention of postoperative adhesion. It can provide the valuable evidence to research the effect of statin on postoperative adhesion for patients having statin due to dyslipidemia. There was a pilot clinical study investigating the effect of oral simvastatin after colorectal surgery^40^. There was no anti-adhesive effect of statin in this study, but further and more clinical trials are required.

The present meta-analysis has several limitations. First, we could not include human clinical studies. The postoperative macroscopic adhesion score, which is the primary end point of this study, cannot be confirmed without operation except in special cases. Thus, for ethical reasons, randomized controlled studies are not possible. Only one study of retrospective data, which examined 419 patients admitted with intraperitoneal adhesion in one institution, reported that history of statin use significantly reduced the need for reoperation^23^.

Secondly, significant heterogeneity was observed between the included studies. Therefore, although our meta-analysis showed that statins are effective for the prevention of postoperative adhesion with respect to gross and microscopic adhesion scores, these results should be cautiously interpreted. However, sensitivity analyses, conducted through the removal of one study at a time, suggest that this had no impact on the statistical significance. Finally, the present meta-analysis included only a small number of available studies. Additional well-designed, large studies will provide the information necessary to further elucidate the issues presented here. Nevertheless, notwithstanding these limitations, we applied rigorous methodology to provide the first systematic review to examine the anti-adhesive effects of statins.

In conclusion, although this meta-analysis does have some limitations, our study demonstrates that statins applied at the surgical area may be effective for the prevention of postoperative adhesion with respect to gross and microscopic adhesion scores. However, clinical studies performed in large patient populations and well-designed large animal studies are required to determine the impact of statins on postoperative adhesion.

## Methods

The present systematic review and meta-analysis was conducted according to the protocol recommended by the Cochrane Collaboration^44^ and reported in accordance with the Preferred Reporting Items for Systematic Reviews and Meta-Analysis (PRISMA) guidelines^45^.

### Literature search

Two authors (GJ Choi and D Lee) independently carried out database searches using OVID-MEDLINE, EMBASE, and Google Scholar in March 2017, which was updated in September 2017. There were no language restrictions in the search criteria. The reference lists of the eligible publications were also searched manually to further identify relevant publications. The search strategy, which included a combination of free text, Medical Subject Headings, and EMTREE terms, is given in detail in the *Appendix*.

### Study selection

The inclusion and exclusion criteria of this study were determined before conducting the systematic search. All animal studies that compared statins applied to the surgical site (statin group) with control group for the prevention of adhesion following surgery were included. Review articles, case reports, case series, letters to the editor, commentaries, proceedings, laboratory science studies, and any other non-relevant studies were excluded. Two authors (HK Park and DS Kim) independently reviewed the titles and abstracts of the reports identified in the search described above. If a report was determined eligible from the title or abstract, the full paper was retrieved. Potentially relevant studies chosen by at least one author were retrieved, and the full-text versions were evaluated. Two authors (HK Park and DS Kim) held discussions to reach consensus on which studies to include. Disagreements over inclusion or exclusion were settled through discussion with the third investigator (H Kang).

### Methodological quality and publication bias

Methodological quality of the selected studies was assessed based on statements of (1) random allocation into treatment and control groups, (2) husbandry conditions (e.g., light/dark cycle, temperature, access to water, and environmental enrichment), (3) compliance with animal welfare regulations, and (4) potential conflicts of interests and whether the study appeared in a peer-reviewed publication. Two authors (GJ Choi and D Lee) independently evaluated the studies and scored each on a scale from 0 to 5. Conflicting evaluations were resolved through the third investigator (H Kang). Publication bias was assessed but not analyzed when the number of included studies was less than 10.

### Outcome measure

We recorded outcomes according to intention to treat analysis where available. The primary outcome measure of this meta-analysis was the severity of adhesion under macroscopic evaluation (gross adhesion). The secondary outcome measure was the severity of fibrosis under microscopic evaluation and t-PA activity.

### Data extraction

All interrelated data from the included studies were independently extracted and entered into standardized forms by two authors (HK Park and DS Kim), and then cross-checked. Discrepancies were resolved through discussion. If an agreement could not be reached, issues were resolved with the aid of a third investigator (D Lee).

We treated the administration of statin at the surgical site as the statin group regardless of its type, dose, or administered method, and we did the administration of placebo and nothing as the control group. We combined all of the statin groups if a given study had more than one statin group which are eligible for comparison^46^. We extracted data from partial groups that were eligible in a study with multiple groups if the groups were comparable. When a material such as film, membrane, and sponge was equally applied or not applied to both statin and control group in a single study with multiple groups more than four, data were extracted to effectively yield to sub-studies of whether the material was used or not. The standardized form included the following items: (1) title, (2) name of first author, (3) name of journal, (4) year of publication, (5) type of animal studied, (6) type of surgery performed, (7) interventions in control group, (8) interventions in experimental group, (9) definition of gross adhesion score, (10) definition of microscopic adhesion score (11) severity and extent of gross adhesion, (12) severity of fibrosis, and (13) t-PA activity.

The data were initially extracted from tables or text. In cases involving missing or incomplete data, an attempt was made to contact the study authors to obtain the relevant information.

### Statistical analysis

We conducted this meta-analysis using the Comprehensive Meta-Analysis software (version 2.0; Biostat, Englewood, NJ, USA). Two authors (GJ Choi and H Kang) independently inputted all data into the software. The standardized mean differences (SMDs) and their 95% confidence intervals (CIs) were calculated for each outcome. We used the chi-squared test for homogeneity and the I^2^ test for heterogeneity. A P < 0.1 for the chi-squared statistic was used to indicate statistical significance. An I^2^ greater than 50% was considered to indicate significant heterogeneity. For P-values < 0.10 and the I^2^ values < 50%, fixed effects models were used, and for I^2^ values > 50%, random effects models were used^47^.

Since the combined number of studies that showed substantial heterogeneity were less than 10, t-statistics (Hartung-Knapp-Sidik-Jonkman method) were used instead of Z-tests in all random effects analysis in order to lower the error rate^48^.

We conducted subgroup analysis according to the surgery type. We also conducted sensitivity analysis on outcomes with significant heterogeneity. If the reported data were medians (P_25_–P_75_), medians (ranges) or means (standard error of means), means and standard deviations were calculated from these values^49^.

## Electronic supplementary material

Appendix

## Data Availability

All data generated or analysed during this study are included in this published article (and its Supplementary Information files).
