# Supplementary material for: Effect of statins on experimental postoperative adhesion: a systematic review and meta-analysis
Source: Sci Rep. 2018 Oct 3;8:14754. doi: 10.1038/s41598-018-33145-z (PMC6170439; doi:10.1038/s41598-018-33145-z)
Supplement: Supplementary file 1 — Appendix [file 41598_2018_33145_MOESM1_ESM.docx]

**Effect of statins on experimental postoperative adhesion: a systematic review and meta-analysis**

Geun Joo Choi^1^, Hee Kyung Park^2^, Dong Su Kim^2^, Donghyun Lee^2,*^, and Hyun Kang^1,*^

^1^ Department of Anesthesiology and Pain Medicine, College of Medicine, Chung-Ang University, 84 Heukseok-ro, Dongjak-gu, Seoul 06911, Republic of Korea

^2^ Department of Biomedical Engineering, School of Integrative Engineering, Chung-Ang University, 84 Heukseok-ro, Dongjak-gu, Seoul 06911, Republic of Korea

**Appendix**

**MEDLINE**

1. Exp General Surgery/
2. surgery.mp.
3. opertation.mp
4. opera$.mp
5. surg$.mp
6. or/1-5
7. exp Tissue Adhesions/
8. adhesion.mp
9. adhesive.mp
10. or/7-9
11. 6 and 10
12. exp Hydroxymethylglutaryl-CoA Reductase Inhibitors/
13. statin.mp
14. or/12-13
15. 11 and 14

**EMBASE**

1. 'general surgery'/exp
2. surgery
3. operation
4. surg$
5. operat$
6. #1 OR #2 OR #3 OR #4 OR #5
7. #1 OR #2 OR #3 OR #4 OR #5 AND [embase]/lim
8. 'adhesion'/exp
9. Adhesion
10. 'adhesion barrier'/exp
11. #8 OR #9 OR #10
12. #7 AND #11
13. 'hydroxymethylglutaryl coenzyme A reductase inhibitor'/exp
14. #13 OR #14
15. #12 AND #15
